# Supplementary figures and images for: Investigating the effects of management practice on mammalian co-occurrence along the West Coast of South Africa
Source: PeerJ. 2020 Jan 27;8:e8184. doi: 10.7717/peerj.8184 (PMC6991126; doi:10.7717/peerj.8184)

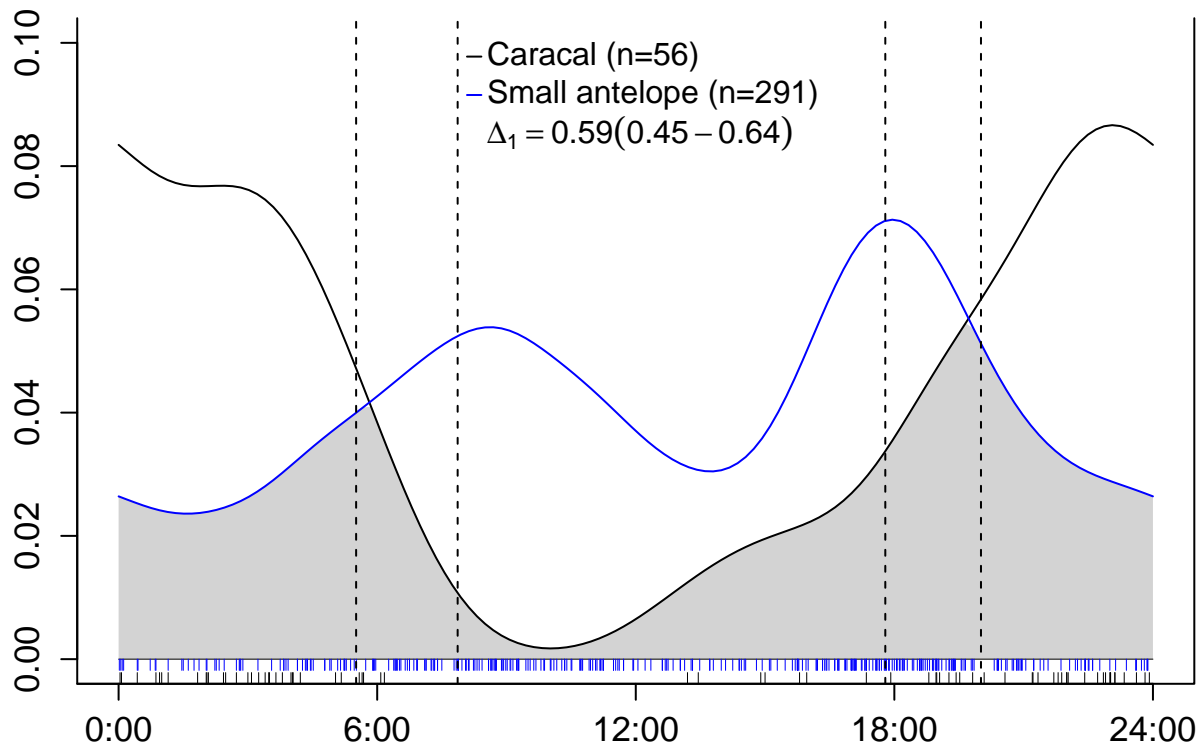

Supplement: Figure S1 — Time of day starting and ending at midnight on the x-axes and the fitted kernel-density on y-axes. The grey shaded area indicates overlap and is described by the coefficient of overlap (Δ) and the associated estimator used (number in subscript) along with the 95% confidence intervals in parentheses. The vertical dotted lines represent the earliest and latest sunrise and sunset times across the study period. ρ is derived based on a Watson-Wheeler test of homogeneity for circular data. The dashed lines along the x-axes indicate the sample size of time-of-day observations. Time of day is displayed on the x-axes with midday in the middle and the fitted kernel-density on y-axis. [file peerj-08-8184-s001.pdf]

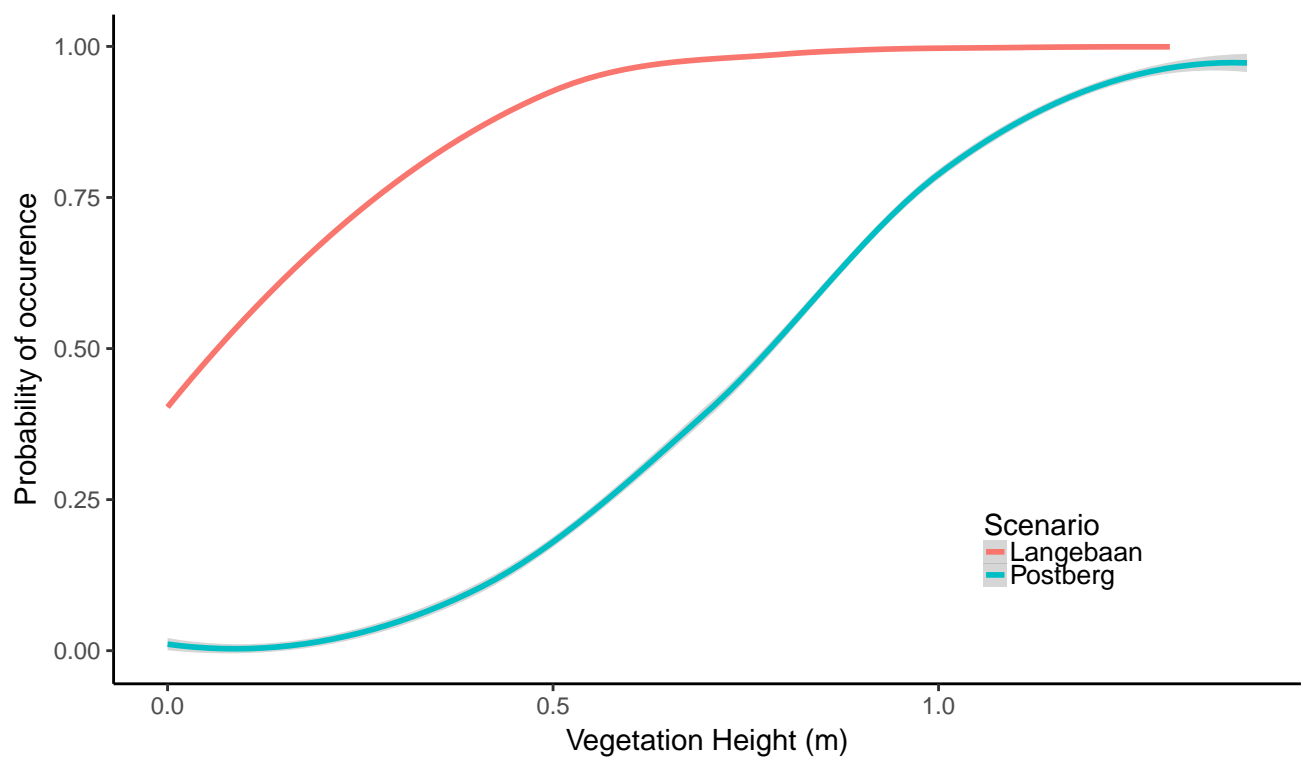

Supplement: Figure S2 — Note that it was not possible to model the effect of covariates on common duiker occurrence in Lambert’s Bay due to 100% occupancy. [file peerj-08-8184-s002.pdf]
