# Supplementary material for: Investigating the effects of management practice on mammalian co-occurrence along the West Coast of South Africa
Source: PeerJ. 2020 Jan 27;8:e8184. doi: 10.7717/peerj.8184 (PMC6991126; doi:10.7717/peerj.8184)
Supplement: Table S1 [file peerj-08-8184-s005.docx]

| **Parameter** | **Description** |
| --- | --- |
| PsiA | Probability of site occupancy of dominant species (managed ungulates). |
| PsiBA | Probability of site occupancy of subordinate species (small antelope), where dominant species (managed ungulates) are present. |
| PsiBa | Probability of site occupancy of subordinate species (small antelope), where dominant species (managed ungulates) are not present. |
| SIF | Species interaction factor: likelihood ratio of co-occurrence, SIF = psiAB/(psiA*psiB) |
