# Supplementary material for: Investigating the effects of management practice on mammalian co-occurrence along the West Coast of South Africa
Source: PeerJ. 2020 Jan 27;8:e8184. doi: 10.7717/peerj.8184 (PMC6991126; doi:10.7717/peerj.8184)
Supplement: Table S2 — Managed ungulates are modelled as the dominant species while small antelope as the subordinate. Parameters are described in Table S1. [file peerj-08-8184-s006.docx]

|  | **psiA** | **SE** | **psiBA** | **SE** | **psiBa** | **SE** | **SIF** | **SE** |
| --- | --- | --- | --- | --- | --- | --- | --- | --- |
| **Langebaan** | | | | | | | | |
| **LU-SA** | 0.957 | 0.055 | 0.942 | 0.056 | NA | NA | 0.997 | 0.004 |
| **LU-CD** | 0.985 | 0.061 | 0.887 | 0.075 | NA | NA | 0.998 | 0.008 |
| **MU-SA** | 0.611 | 0.123 | 0.909 | 0.087 | NA | NA | 0.963 | 0.040 |
| **MU-CD** | 0.579 | 0.182 | 0.808 | 0.130 | NA | NA | 0.909 | 0.086 |
| **Lamberts Bay** | | | | | | | | |
| **LV-SB** | 0.286 | 0.109 | 0.809 | 0.176 | 0.921 | 0.173 | 0.910 | 0.195 |
