# Supplementary material for: Investigating the effects of management practice on mammalian co-occurrence along the West Coast of South Africa
Source: PeerJ. 2020 Jan 27;8:e8184. doi: 10.7717/peerj.8184 (PMC6991126; doi:10.7717/peerj.8184)
Supplement: Supplemental Information 1 [file peerj-08-8184-s007.docx]

Supplementary material

Two-species occupancy results

Langebaan

Large herbivore occupancy estimates (psiA) were high as determined by interaction models with small antelope (0.957 ± 0.055 SE) and common duiker (0.985 ± 0.061 SE). Small antelope (0.942 ± 0.056 SE) and common duiker (0.887 ± 0.075 SE) had high probability of occurrence in the presence of large herbivores (psiBA). The species interaction factor (SIF) between large ungulates was close to one for both small antelope (0.997 ± 0.004 SE) and common duiker (0.998 ± 0.008 SE) (Table 3).

Medium ungulate occupancy estimates (psiA) in the interaction models with small antelope (0.611 ± 0.123 SE) and common duiker (0.579 ± 0.182 SE) were lower than the estimates for large ungulates. Small antelope (0.909 ± 0.087 SE) and common duiker (0.808 ± 0.130 SE) however, still had high probability of occurrence in the presence of medium sized ungulates (psiBA). The SIF between medium ungulates and both small antelope (0.963 ± 0.040 SE) and common duiker was also close to one (0.909 ± 0.086 SE) (Table 3).

Lamberts Bay (farm)

The only viable interaction model for the farm was between livestock and steenbok where livestock probability of occurrence (psiA) was 0.286 (± 0.109 SE). The probability of steenbok occurring in the presence of livestock (psiBA) was 0.809 (± 0.176 SE) while in the absence (psiBa), it was 0.921 (± 0.173 SE). Similar to the Langebaan site, SIF between livestock and steenbok was close to one (0.910 ± 0.195 SE) (Table 3).

Species interaction Factors (SIF) close to one are indicative of species that occur independently of one another, whereas SIF > 1 indicates species occur together more frequently than expected (facilitation) and SIF < 1 indicates that species occur together less often than expected (competition) (ref). As such, our results suggest that small and large antelope occur independently of one another across our study sites.
